# Clinical value of TAT, PIC and t-PAIC as predictive markers for severe sepsis in pediatric patients

**DOI:** 10.3389/fped.2024.1336583

**Published:** 2024-03-18

**Authors:** Huan Bai, Ling Shen, Hailong Zhang, Ning Tang

**Affiliations:** ^1^ Department of Laboratory Medicine, Tongji Hospital, Tongji Medical College, Huazhong University of Science and Technology, Wuhan, China; ^2^ Department of Laboratory Medicine, Shanxi Bethune Hospital, Shanxi Academy of Medical Sciences, Tongji Shanxi Hospital, Third Hospital of Shanxi Medical University, Taiyuan, China

**Keywords:** pediatric, severe sepsis, thrombin-antithrombin complex (TAT), α2-plasmininhibitor- plasmin complex, tissue-type plasminogen activator-inhibitor complex

## Abstract

**Objective:**

Sepsis in pediatric patients can progress to severe sepsis, and identifying biomarkers of this progression may permit timely intervention to prevent it. This study aimed to investigate the ability of thrombin-antithrombin complex (TAT), α2-plasmininhibitor-plasmin complex (PIC) and tissue-type plasminogen activator-inhibitor complex (t-PAIC) to predict severe sepsis in pediatrics early.

**Methods:**

148 eligible pediatric sepsis patients were enrolled in this study, and were then divided into those who progressed to severe sepsis (*n* = 50) or not (*n* = 98). Serum levels of TAT, PIC, and t-PAIC were analysed, and simplified pediatric critical illness score (PCIS) and DIC score were calculated on the day of pediatric sepsis diagnosis.

**Results:**

Compared with sepsis patients, severe sepsis patients had higher levels of TAT, PIC and t-PAIC. Correlation analysis revealed that TAT, PIC and t-PAIC were significantly correlated with simplified PCIS and DIC score. ROC curve analysis suggested that TAT, PIC and t-PAIC could serve as biomarkers for predicting severe sepsis with the AUC up to 0.862, 0.759 and 0.851, respectively. Stratified analysis demonstrated that the patients with increased levels of TAT, PIC and t-PAIC had worse illness severity and clinical outcome. Univariate logistic regression analysis revealed that TAT, PIC and t-PAIC were all risk factors for severe sepsis, yet only TAT and t-PAIC were independent risk factors in multivariate model.

**Conclusions:**

TAT, PIC and t-PAIC could serve as biomarkers for predicting severe sepsis, and correlated with illness severity in pediatrics, what's more, serum levels of TAT and t-PAIC may be independent risk factors for pediatric severe sepsis.

## Introduction

Pediatric sepsis was thought to be a clinical syndrome characterized by systemic inflammatory response syndrome (SIRS) secondary to infection over the years (1), much lately, a new version of “International Consensus Criteria for Pediatric Sepsis and Septic Shock” had been proposed, and pediatric sepsis was redefined as the dysregulation of the body's response to infection, leading to life-threatening organ dysfunction (2). The updated guideline indicates a growing understanding of the nature of pediatric sepsis. It is worth mentioning that this syndrome is among the primary causes why children are admitted to the pediatric intensive care unit (PICU) (3). The term “severe sepsis” emphasizes tissue infiltration and/or multiple organ dysfunction followed by sepsis, and septic shock is a special type of severe sepsis that is accompanied by cardiovascular organ dysfunction (1). Sepsis in pediatric patients can progress to severe sepsis, and this progression is related to a rise in the mortality rate from 1%–5% for sepsis to 9%–20% for severe sepsis among pediatrics (4). Hence, early identification of this progression may enable more efficient and timely intervention.

The onset, progression and outcome of sepsis are frequently associated with coagulation abnormalities, and the excessive crosstalk between inflammation and coagulation plays a vital role in these pathogenesis, which includes dysfunction of clotting cascade process, anticoagulant and fibrinolytic systems, together with related endothelial damage (5–7). Sepsis-related coagulopathy can range in severity from mildly decreased platelet counts and prolonged clotting time, which are indicative of subclinical issues, to more severe coagulopathies such as disseminated intravascular coagulation (DIC) (7, 8). Classic coagulation laboratory tests, such as activated partial thromboplastin time (APTT), prothrombin time (PT), and platelet count, mainly indicate signs of consumption and impaired synthesis instead of ongoing coagulopathy, and change slowly in the disease course (9, 10), and as a result, they might not be particularly available for identifying coagulopathy in the early stages of a patient's illness, which makes therapy monitoring difficult among sepsis patients. Thus, it's necessary to further investigate more specific or sensitive molecular markers.

Thrombin-antithrombin complex (TAT), α2-plasmininhibitor-plasmin complex (PIC) and tissue-type plasminogen activator-inhibitor complex (t-PAIC) are biomarkers of thrombin generation, fibrinolysis and endothelium injury (10, 11), respectively, which may shed light on the pathophysiology of sepsis-associated coagulopathy from various perspectives. TAT, a molecular complex consisting of thrombin and antithrombin, is thought to be a sensitive indicator of coagulation activation and thrombin production, both of which are essential for sepsis-associated coagulopathy (12). PIC is a molecular complex composed of plasmin and α2-plasmin inhibitor, and is considered to be a sign of plasmin formation and fibrinolysis activation (13). t-PAIC is a marker of endothelial injury and fibrinolysis activation, which is formed through the combination of tissue plasminogen activator and plasminogen activator inhibitor-1 (PAI-1), and is thought to be related with organ failure due to extensive microthrombosis (14). Hence, these biomarkers could serve as highly sensitive indicators of coagulation and fibrinolysis, and may highlight even slight hemostatic alterations, which might potentially provide crucial cues for prompt and effective intervention in sepsis patients.

Previous studies have explored the clinical utilities of TAT, PIC and t-PAIC mainly focused on adult population (11, 15–17), however, there were little researches concerned about pediatrics. In this study, we aimed to investigate whether serum levels of TAT, PIC, and t-PAIC could act as early warning signs of severe sepsis in pediatric patients.

## Materials and methods

### Study design and participants

This single center, observational study was conducted in Tongji Hospital, Tongji Medical College, Huazhong University of Science and Technology (Wuhan, China), which was the biggest tertiary hospital in Central China. Eligible pediatric patients who were diagnosed with sepsis in PICU between November 2020 and November 2021 were enrolled. The diagnosis of pediatric sepsis was according to the criteria of 2005 International Pediatric Sepsis Consensus Conference, which emphasized SIRS plus suspected or proven infection (1). Exclusion criteria included: age younger than 28 days or older than 18 years, clinical conditions complicated with hematological malignancy, severe hepatic and (or) renal insufficiency, severe immunodeficiency, severe trauma or other acute coagulation challenges, and so on. Pediatric severe sepsis were then diagnosed if they met any one condition of below: cardiovascular organ dysfunction or acute respiratory distress syndrome or two or more other organ dysfunctions (1). Additionally, 20 healthy controls (HCs) with matched age and sex were recruited and identified through physical examination and interview.

This study was approved by the ethical committee of Tongji Hospital, Tongji Medical College, Huazhong University of Science and Technology, and all of the participants' legal guardians or next of kin provided written informed consent for their participation of the study (TJ-IRB20210953).

### Data collection

Demographic and clinical data including sex, age, site of infection, illness severity and clinical outcome were retrieved from our hospital's electronic medical records. The following indicators were collected on the initial day of pediatric sepsis diagnosis: APTT, PT, international normalized ratio (INR), thrombin time (TT), fibrinogen, D-dimer, fibrin (and/or fibrinogen) degradation products (FDP), antithrombin III, and platelet count. In addition, the levels of TAT, PIC and t-PAIC were analysed at the same time. Simplified pediatric critical illness score (PCIS) is a widely used scoring tool assessing pediatric illness severity in China, which includes 8 easily accessible clinical and laboratory indicators, as listed below: respiratory rate, blood pressure, heart rate, blood potassium, blood sodium, urea nitrogen or blood creatinine, hemoglobin, gastrointestinal system symptoms (bleeding from stress ulcer or intestinal paralysis). The total score is 80 points, and the lower the score, the worse severity of the illness (18). DIC score followed the diagnostic criteria of the International Society on Thrombosis and Haemostasis (ISTH) (19). The scores of simplified PCIS and DIC were calculated in the PICU upon initial diagnosis of pediatric sepsis. The levels of APTT, PT, INR, TT, fibrinogen, D-dimer, FDP and antithrombin III were determined by STA R MAX coagulation analyzer (STAGO, Paris, France), the count of platelet was analysed by XN-9000 automatic hematology analyzer (SYSMEX, Kobe, Japan), and the levels of TAT, PIC and t-PAIC were assayed by HISCL 5000 analyser with original chemiluminescence reagents (SYSMEX, Kobe, Japan).

### Statistical analysis

Continuous variables were displayed as mean ± standard deviation (SD) or median with interquartile range (IQR) accorded with normal or abnormal distribution, as appropriate. Categorical variables were displayed as *n* (%). Event frequencies were compared with chi-squared test. Continuous variables were compared with independent-samples *T* test or Mann-Whitney *U*-test, as appropriate. Pearson or spearman' s rank correlation test for normal distribution or non-normal distribution continuous data were performed to explore the relationship between two variables, as appropriate. Receiver operating characteristic (ROC) curve analysis was conducted to determine the optimal cutoff values linked to highest sensitivity and specificity. Univariate or multivariable logistic regression were applied to explore risk factors for pediatric severe sepsis. Statistical analyses were performed using GraphPad Prism version 8 (San Diego, CA, USA) and SPSS version 22.0 (SPSS, Chicago, IL, USA). A significant level of *p* < 0.05 was established for statistical analysis.

## Results

### Characteristics of the subjects

**Figure 1:**
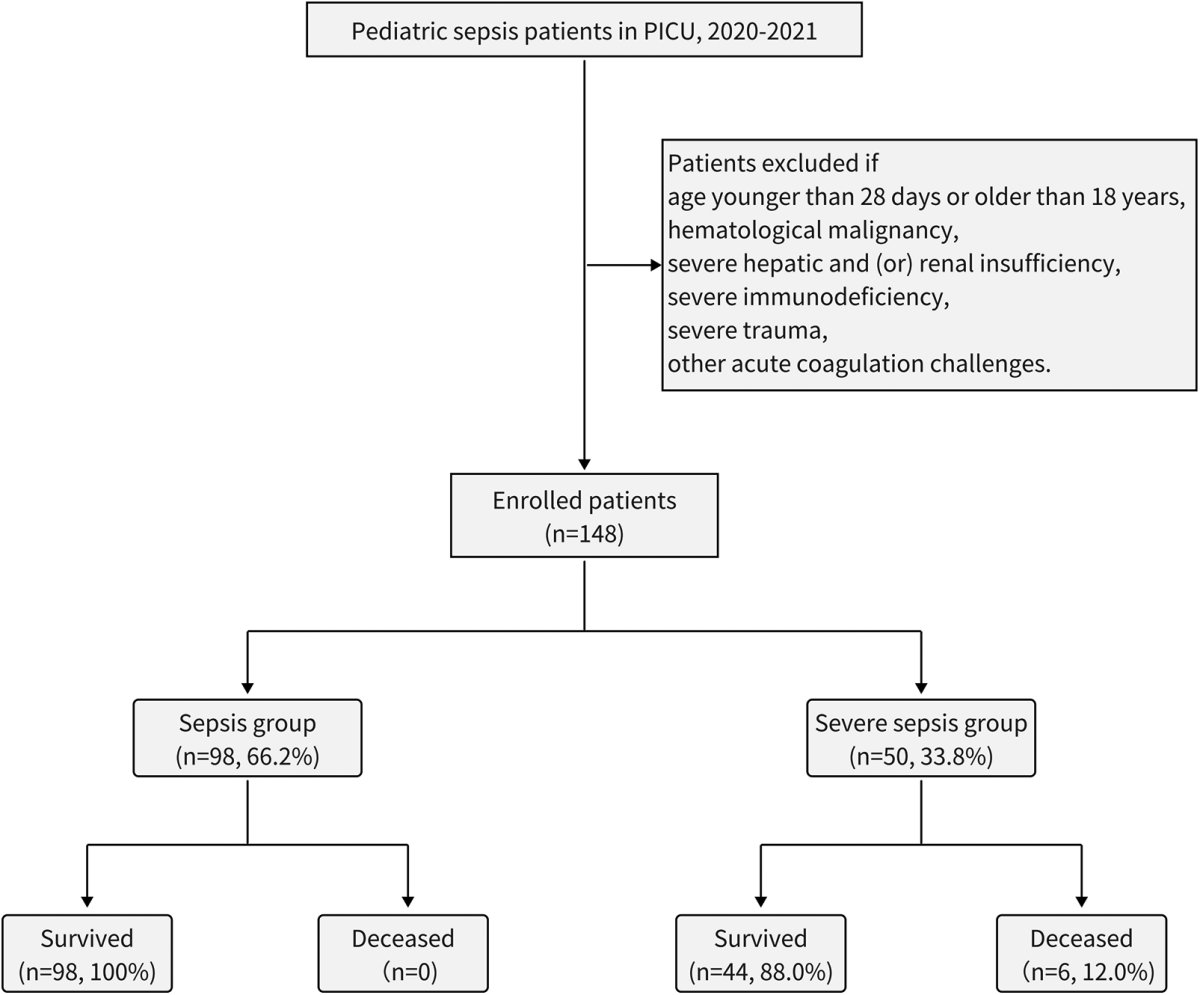
Patient flow diagram.

The present study enrolled 148 pediatric patients diagnosed with sepsis, including 90 (60.8%) males and 58 (39.2%) females, median age of 2.58 years (25th–75th quartiles, range 1.00–4.27 years), of whom 50 (33.8%) were diagnosed with severe sepsis during the stays of PICU. The most common site of infection was respiratory organs (66.9%), followed by urinary organs (8.8%) and digestive organs (5.4%). Of those diagnosed with severe sepsis, 6 (12.0%) died (Figure 1). The basic clinical characteristics of the subjects are presented in Table 1.

**Table 1:** Baseline characteristics between sepsis and severe sepsis in pediatric patients.

	HCs (*n* = 20)	Overall (*n* = 148)	Sepsis (*n* = 98)	Severe sepsis (*n* = 50)
Sex, male/female	12/8	90/58	64/34	26/24
Age, years	3.04 (1.25, 4.79)	2.58 (1.00, 4.27)	2.58 (0.81, 4.12)	2.42 (1.23, 4.71)
Site of infection, *n*(%)				
Respiratory		99 (66.9)	60 (61.2)	39 (78.0)***
Urinary		13 (8.8)	12 (12.2)	1 (2.0)
Digestive		8 (5.4)	6 (6.1)	2 (4.0)
Other/unknown		28 (18.9)	20 (20.4)	8 (16.0)
Classical coagulation tests				
PT, s	12.8 ± 0.5	14.4 ± 1.5**	14.5 ± 1.29	14.2 ± 1.79
INR	0.97 ± 0.05	1.13 ± 0.15**	1.14 ± 0.13	1.11 ± 0.18
APTT, s	38.3 ± 3.1	45.9 ± 8.7**	45.8 (41.2, 51.9)	43.7 (37.0, 51.4)
Fibrinogen, g/L	2.66 ± 0.51	3.87 (2.80, 5.29)**	4.16 (3.34, 5.65)	3.04 (2.11, 4.58)*****
TT, s	17.7 ± 0.8	15.9 (15.2, 17.6)*	15.7 (14.9, 16.7)	17.7 (15.7, 19.9)*****
D-dimer, ug/ml	0.26 (0.22, 0.38)	0.80 (0.39, 2.43)**	0.52 (0.31, 1.01)	2.63 (1.33, 5.62)*****
FDP, ug/ml	4.00 (4.00, 4.00)	4.00 (4.00, 6.98)*	4.00 (4.00, 4.11)	8.37 (4.00, 17.15)*****
Antithrombin III, %	108.7 ± 10.8	92.3 ± 22.2**	91.5 (82.8, 103.0)	94.0 (74.5, 108.3)
Platelet count, ×10^9^/L	317 ± 80	281 ± 135	289 (237, 387)	190 (107, 323)*****
Specific coagulation markers				
TAT, ng/ml	2.05 (1.45, 2.35)	3.55 (2.00, 7.90)*	2.55 (1.60, 4.23)	9.65 (6.98, 19.75)*****
PIC, ug/ml	0.52 (0.42, 0.61)	0.91 (0.57, 1.51)**	0.78 (0.51, 1.09)	1.59 (0.95, 2.88)*****
TAT/PIC ratio	3.98 (2.55, 5.45)	4.49 (2.30, 7.62)	3.54 (1.88, 6.05)	6.91 (4.10, 10.72)*****
t-PAIC, ng/ml	2.50 (1.90, 2.90)	3.70 (2.30, 7.25)*	2.90 (2.10, 4.43)	8.95 (4.30, 18.10)*****
Illness severity				
Use of vasopressor, *n* (%)		8 (5.4)	0	8 (16.0)*****
Mechanical ventilation, *n* (%)		38 (25.7)	14 (14.3)	24 (48.0)*****
Number of organ dysfunction		1 (0, 2)	0.5 (0, 1)	2 (2, 4)*****
DIC, *n* (%)		36 (24.3)	6 (6.1)	30 (60.0)*****
DIC score		1 (0, 2)	0 (0, 1.25)	3 (2, 4)*****
Simplified PCIS score		72 (70, 76)	74 (72, 76)	69 (64, 72)*****
Outcome				
Mortality, *n* (%)		6 (4.1)	0	6 (12.0)****
PICU stay, days		6 (4, 10)	5 (3, 7)	10 (6, 12)*****
Total hospital stay, days		11 (7, 17)	10 (7, 14)	18 (12, 22)*****

PT, prothrombin time; INR, international normalized ratio; APTT, activated partial thromboplastin time; TT, thrombin time; FDP, fibrin (and/or fibrinogen) degradation products; TAT, thrombin-antithrombin complex; PIC, α2-plasmininhibitor-plasmin complex; t-PAIC, tissue-type plasminogen activator-inhibitor complex; DIC, disseminated intravascular coagulation; PCIS, pediatric critical illness score; PICU, pediatric intensive care unit; HCs, healthy controls. Use of vasopressor referred to the use of dopamine, dobutamine, epinephrine, and/or norepinephrine.

* *p *< 0.01, compared with HCs.

** *p *< 0.001, compared with HCs.

*** *p *< 0.05, compared with sepsis group.

**** *p *< 0.01, compared with sepsis group.

***** *p *< 0.001, compared with sepsis group; Data were expressed as median (interquartile range), mean ± SD, or no. (%).

**Figure 2:**
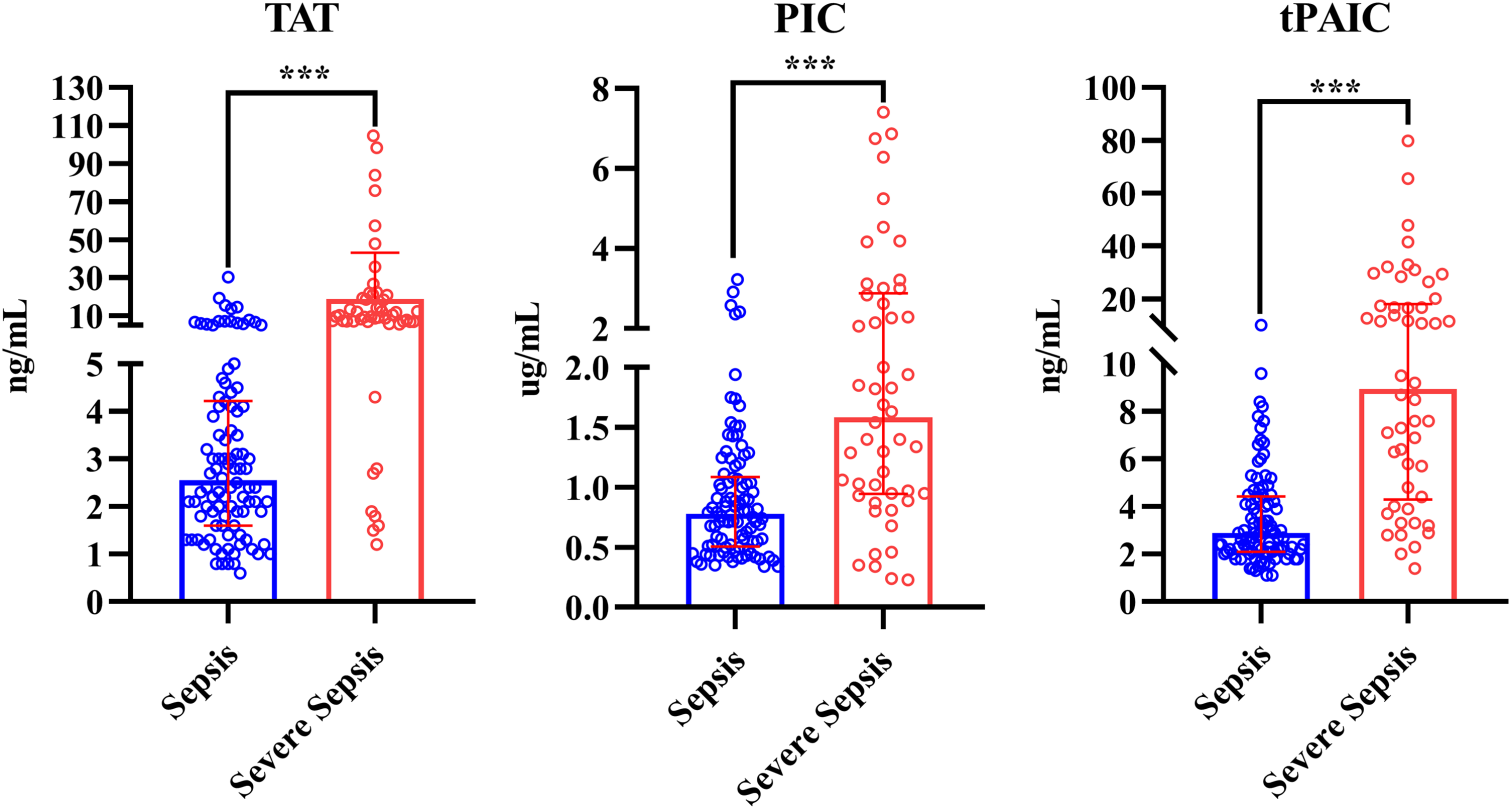
Comparison of TAT, PIC and t-PAIC between pediatric patients with sepsis and severe sepsis groups. TAT, thrombin-antithrombin complex; PIC, α2-plasmininhibitor-plasmin complex; t-PAIC, tissue-type plasminogen activator-inhibitor complex; ***p < 0.001.

Compared with HCs, overall pediatric sepsis patients had significant longer PT, APTT, but shorter TT, higher INR, fibrinogen, D-dimer and FDP level, yet lower antithrombin III level, in addition, the levels of TAT, PIC and t-PAIC were significantly increased in overall pediatric sepsis patients (Table 1). Based on the illness severity, the overall pediatric sepsis patients were then separated into two groups: sepsis group and severe sepsis group according to the criteria of 2005 International Pediatric Sepsis Consensus Conference (1). Severe sepsis group patients showed longer TT, less fibrinogen level, higher D-dimer and FDP levels, and lower platelet count than sepsis group patients. What is more, the levels of TAT, PIC and t-PAIC were significantly increased in severe sepsis group patients, when compared with sepsis group patients (Table 1 and Figure 2). Regarding for the illness severity and clinical outcome, severe sepsis group patients showed higher rates of the use of vasopressor, mechanical ventilation, DIC occurrence and mortality, more numbers of organ dysfunction, higher score of DIC, yet lower score of simplified PCIS, and longer time of PICU stay and total hospital stay (Table 1).

### Correlation analysis between TAT, PIC and t-PAIC and clinical severity score

Spearman's rank correlation analysis demonstrated that TAT, PIC and t-PAIC were positively correlated with DIC score, and negatively correlated with simplified PCIS, which suggested these three biomarkers were all significantly correlated with the severity of the disease (Table 2).

**Table 2:** Correlation analysis between specific coagulation markers and clinical severity score.

	Simplified PCIS score		DIC score	
	*r* value	*p* value	*r* value	*p* value
TAT	−0.406	<0.001	0.421	<0.001
PIC	−0.174	0.034	0.571	<0.001
t-PAIC	−0.423	<0.001	0.515	<0.001

TAT, thrombin-antithrombin complex; PIC, α2-plasmininhibitor-plasmin complex; t-PAIC, tissue-type plasminogen activator-inhibitor complex; PCIS, pediatric critical illness score; DIC, disseminated intravascular coagulation.

### Ability of TAT, PIC and t-PAIC to predict severe sepsis in pediatrics

**Figure 3:**
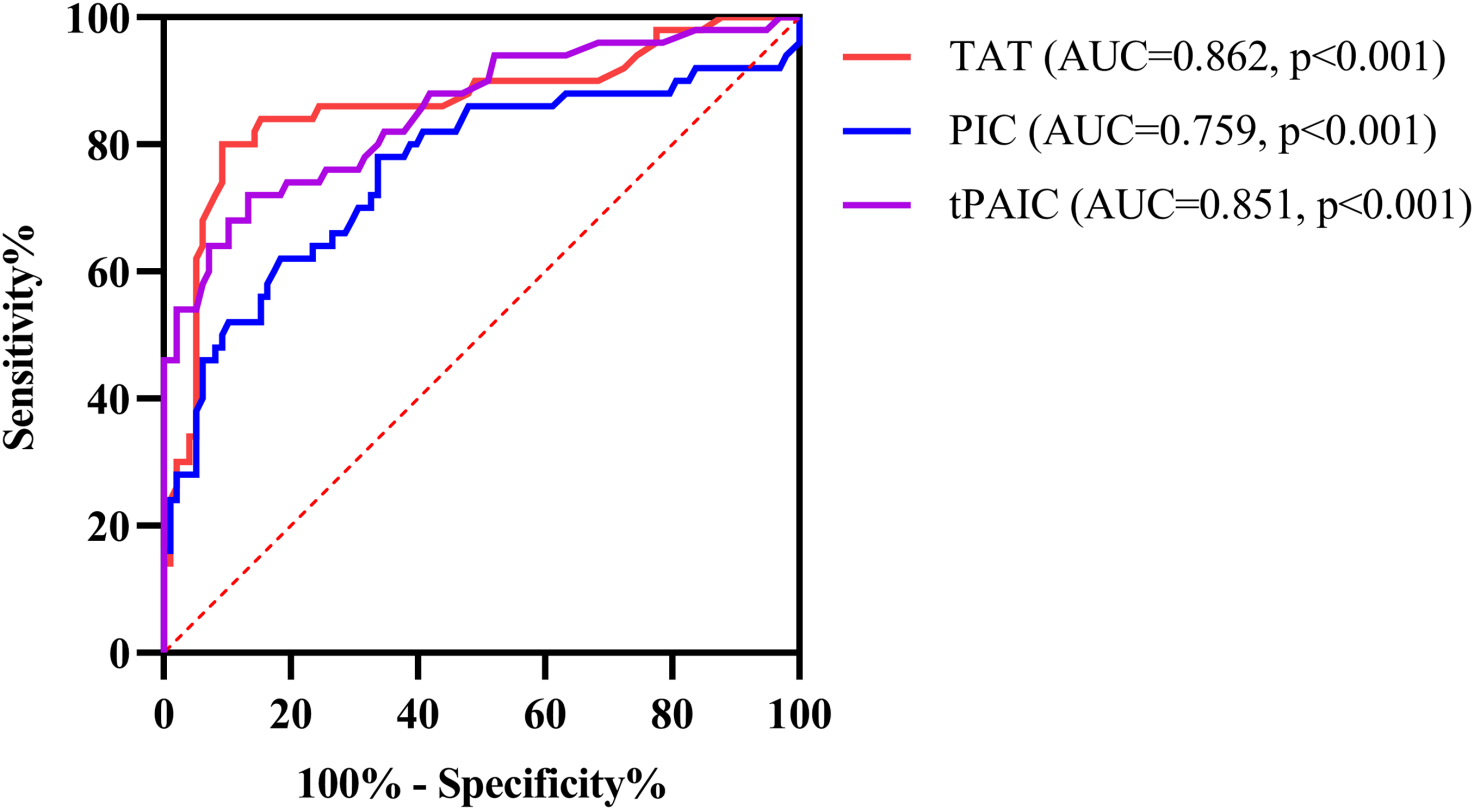
ROC curve analysis of TAT, PIC and t-PAIC in predicting severe sepsis in pediatric patients. TAT, thrombin-antithrombin complex; PIC, α2-plasmininhibitor-plasmin complex; t-PAIC, tissue-type plasminogen activator-inhibitor complex; ROC, receiver operator characteristic; AUC, area under the curve.

ROC curve analysis showed that the area under the curve (AUC) for TAT, PIC and t-PAIC to predict severe sepsis among 148 pediatric sepsis patients were 0.862, 0.759, and 0.851, respectively, with the optimal cut-off values of 6.85 ng/ml, 0.92 ug/ml and 5.5 ng/ml, respectively, which gave the sensitivity of 80.0%, 78.0% and 72%, respectively, and the specificity of 90.8%, 66.3%, and 86.7%, respectively (Table 3 and Figure 3).

**Table 3:** Predictive efficiency of TAT, PIC and t-PAIC in pediatric patients with severe sepsis.

	AUC	Cut-off value	Sensitivity	Specificity	PPV	NPV	Odds ratio
TAT	0.862	6.85	80.0%	90.8%	81.6%	89.9%	39.556
PIC	0.759	0.92	78.0%	66.3%	54.2%	85.5%	6.983
t-PAIC	0.851	5.5	72.0%	86.7%	73.5%	85.9%	16.813

AUC, area under the receiver operating characteristic curves; PPV, positive predictive value; NPV, negative predictive value; TAT, thrombin-antithrombin complex; PIC, α2-plasmin inhibitor- plasmin complex; t-PAIC, tissue plasminogen activator-inhibitor complex.

### Differences in disease severity and clinical outcome between patients stratified by TAT, PIC and t-PAIC

Based on the optimal cut-off values of TAT (6.85 ng/ml), PIC (0.92 ug/ml) and t-PAIC (5.5 ng/ml), derived from ROC curve analysis, the 148 pediatric sepsis patients were then stratified into two clusters. Stratified analysis showed that those at or above this level of TAT, PIC or t-PAIC had significantly higher rates of severe sepsis and DIC occurrence, longer days of PICU stay and total hospital stay, higher score of DIC, yet lower score of simplified PCIS, and more numbers of organ dysfunction. In addition, patients with higher level of TAT showed higher rates of mechanical ventilation, and those with higher level of t-PAIC had more mortality rate and use of vasopressor (Table 4).

**Table 4:** Comparison of clinical indicators based on the stratification of specific coagulation markers.

	TAT (ng/ml)		PIC (ug/ml)		t-PAIC (ng/ml)	
	<6.85 (*n* = 99)	≥6.85 (*n* = 49)	<0.92 (*n* = 76)	≥0.92 (*n* = 72)	<5.5 (*n* = 99)	≥5.5 (*n* = 49)
Severe sepsis, *n* (%)	10 (10.1)	40 (81.6)***	11 (14.5)	39 (54.2)***	14 (14.1)	36 (73.5)***
Mortality, *n* (%)	2 (2.0)	4 (8.2)^a^	3 (3.9)	3 (4.2)^a^	1 (1.0)	5 (10.2)*
DIC, *n* (%)	8 (8.1)	28 (57.1)***	8 (10.5)	28 (38.9)***	7 (7.1)	29 (59.2)***
Use of vasopressor, *n* (%)	4 (4.0)	4 (8.2)^a^	3 (3.9)	5 (6.9)^a^	2 (2.0)	6 (12.2)*
Mechanical ventilation, *n* (%)	16 (16.2)	22 (44.9)***	15 (19.7)	23 (31.9)^a^	22 (22.2)	16 (32.7)^a^
PICU stay, days	5 (3, 8)	9 (5, 12)***	5 (3, 8)	7 (4, 11)*	5 (3, 7)	9 (6, 12)***
Total hospital stay, days	10 (7, 15)	15 (11, 20)***	10 (7, 16)	13 (9, 19)*	9 (7, 15)	16 (12, 21)***
simplified PCIS score	74 (70, 76)	70 (66, 72)***	72 (70, 76)	72 (66, 76)*	72 (70, 76)	70 (65, 72)***
DIC score	0 (0, 2)	3 (2, 4)***	0 (0, 1)	2 (0, 3)***	0 (0, 2)	3 (2, 3.5)***
Number of organ dysfunction	1 (0, 1)	2 (2, 3.5)***	1 (0, 1)	2 (0, 3)***	1 (0, 1)	2 (1, 4)***

TAT, thrombin-antithrombin complex; PIC, α2-plasmininhibitor-plasmin complex; t-PAIC, tissue-type plasminogen activator-inhibitor complex; DIC, disseminated intravascular coagulation; PICU, pediatric intensive care unit; PCIS, pediatric critical illness score. Use of vasopressor referred to the use of dopamine, dobutamine, epinephrine, and/or norepinephrine.

Data were expressed as median (interquartile range) or no. (%).

^a^ ns, not significant.

* *p *< 0.05.

** *p *< 0.01.

*** *p *< 0.001.

### Independent risk factors for severe sepsis in pediatrics

The univariate and multivariable logistic regression analysis were performed in 148 pediatric sepsis patients for identifing the independent risk factors for severe sepsis. The univariate analysis identified the following variables associated with severe sepsis: fibrinogen, TT, D-dimer, FDP, platelet count, TAT, PIC and t-PAIC. Furthermore, multivariable logistic analysis revealed that only TT, D-dimer, TAT, and t-PAIC were independent risk factors for severe sepsis in pediatrics (Table 5).

**Table 5:** Uni- and multivariate analysis to identify risk factors for severe sepsis in pediatric patients.

	Univariate		Multivariate	
	Odds ratio (95% CI)	*p* value	Odds ratio (95% CI)	*p* value
PT (s)	0.863 (0.678, 1.097)	0.229	/	/
INR	0.238 (0.022, 2.580)	0.238	/	/
APTT (s)	0.971 (0.932, 1.011)	0.150	/	/
fibrinogen (g/L)	0.622 (0.485, 0.797)	<0.001	/	0.095
TT (s)	1.658 (1.327, 2.072)	<0.001	1.455 (1.027, 2.061)	0.035
D-dimer (ug/ml)	2.179 (1.594, 2.979)	<0.001	1.515 (1.105, 2.076)	0.010
FDP (ug/ml)	1.280 (1.150, 1.424)	<0.001	/	0.578
antithrombin III (%)	0.995 (0.980, 1.011)	0.522	/	/
platelet count (×10^9^/L)	0.994 (0.991, 0.997)	<0.001	/	0.554
TAT (ng/ml)	1.277 (1.158, 1.409)	<0.001	1.113 (1.012, 1.223)	0.028
PIC (ug/ml)	3.271 (1.926, 5.557)	<0.001	/	0.696
t-PAIC (ng/ml)	1.530 (1.296, 1.806)	<0.001	1.320 (1.110, 1.569)	0.002

PT, prothrombin time; INR, international normalized ratio; APTT, activated partial thromboplastin time; TT, thrombin time; FDP, fibrin (and/or fibrinogen) degradation products; TAT, thrombin-antithrombin complex; PIC, α2-plasmininhibitor-plasmin complex; t-PAIC, tissue-type plasminogen activator-inhibitor complex; CI, confidence interval.

## Discussion

This single center, observational study offers novel evidence that TAT, PIC, and t-PAIC may be useful biomarkers for the early prediction of severe sepsis in pediatrics, among which, TAT and t-PAIC may be independent risk factors for the development of such severe symptom. We found significantly higher levels of TAT, PIC and t-PAIC in pediatrics with severe sepsis than in those with sepsis, and the levels correlated positively with DIC score, and negatively with simplified PCIS. ROC curve analysis identified the optimal cut-off values of 6.85 ng/ml, 0.92 ug/ml and 5.5 ng/ml for TAT, PIC and t-PAIC, respectively, in predicting severe sepsis among pediatrics. Patients above each threshold had significantly worse illness severity and clinical outcome. According to our findings, the assaying of TAT, PIC, and t-PAIC in pediatric sepsis may aid in the screening of patients who are at higher risk of developing severe sepsis, allowing for prompt management that may lessen or even eliminate the condition.

Severe sepsis dramatically increases the risk of mortality in pediatric sepsis (4), as our cohort showed, where the mortality rate for patients with severe sepsis was 12.0% but for patients with sepsis was zero. In sepsis, the excessive crosstalk between inflammation and coagulation plays a crucial role in the pathogenesis of this clinical syndrome, as to say in detail, inflammation induces coagulation activation but coagulation also markedly influences inflammatory activity (5–7). Massive release of inflammatory cytokines in sepsis can injury vascular endothelial barrier, and induce the expression of tissue factor, which then initiates the coagulation activation (6, 7). As the disease progresses, microvascular thrombosis is constantly forming, however, the clearance is hindered due to anticoagulation impairment and insufficient fibrinolysis in sepsis, eventually leading to tissue and organ hypoperfusion, multiple organ dysfunction syndrome, DIC and (or) even death (20, 21).

Precisely identifying the patient's coagulation disorder is crucial for the management of pediatric sepsis. As discussed above, microthrombus formation in the early stage of sepsis is considered to be immune or protective microthrombus, which could bind pathogens, restricting their spread in the body (22). Hence, if anticoagulant treatment initiates at this time, instead of improving the condition, it may be harmful for the patient. With the progression of the disease, pathogens and inflammation cause massive damage to the endothelium and continuous production of thrombin, which eventually leads to continuous formation of microthrombus (5–7), anticoagulant therapy seems necessary at this stage, however, when and how to grasp the timing of anticoagulation still remains a big clinical challenge. In 2017, ISTH first proposed the concept of sepsis-induced coagulopathy (SIC) and established the SIC scoring system, aiming at identifing the “sweet-spot” for initiating anticoagulant therapy (23). Although, the efficacy of SIC scoring system had been confirmed by some researches based on adults (24–27), however, whether it can be used to guide anticoagulation therapy in pediatrics with sepsis still needs more clinical studies. In addition, DIC, as the most serious type of coagulation disorder in sepsis, is commonly found in patients with severe sepsis or septic shock (28). Early recognition of sepsis DIC and timely management of such fatal coagulation disorder are of great significance in the aspect of improving the condition and reducing the mortality (28).

As mentioned above, coagulation abnormalities and endothelium injury are essential components in the pathophysiology of sepsis (5–7). TAT, PIC and t-PAIC are sensitive biomarkers of coagulation dysfunction and (or) endothelium injury (12–14), which could be served as potential indicators to precisely identify the patient's coagulation disorder. TAT, a molecular complex consisting of thrombin and antithrombin, is thought to be a sensitive indicator of coagulation activation and thrombin production (12). Our study suggested that it was common in pediatric sepsis to be along with coagulation activation and thrombin formation, and the amount of thrombin formation was closely related to the severity of the disease. Koyama K et al. (16) carried out a prospective observational analysis in a single adult intensive care unit, and assayed 14 plasma biomarkers including global markers, markers of thrombin generation, markers of anticoagulants, markers of fibrinolysis, and a marker of endothelial activation among 77 adult sepsis patients, demonstrating that 98.7% TAT, 88.3% protein C and 97.4% FDP had hemostatic abnormalities at baseline, which indicated that coagulopathy had already existed in the very early stages of sepsis. Of the 14 plasma biomarkers that were evaluated, TAT, PAI-1, and protein C activity on ICU admission were the most efficient combination to identify patients who had overt DIC from those who didn't, with an AUC of up to 0.95. This suggested that sepsis patients with overt DIC displayed characteristic traits of coagulation activation, fibrinolytic inhibition, and anticoagulation deficiency, in addition, regarding for the short-term death prediction, only TAT and PAI-1 were found to be significant indicators of 28-day mortality, which was similar to our findings. It is worth mentioning that excessive formation of thrombin, especially combined with anticoagulation impairment and fibrinolytic inhibition, can lead to tissue hypoperfusion and multiple organ dysfunction, which is significantly associated with poor prognosis (20, 21). PIC is a molecular complex composed of plasmin and *α*2-plasmin inhibitor, and is considered to be a sign of plasmin formation and fibrinolysis activation (13). In sepsis, as coagulation initiates, fibrinolysis is then activated, forming a set of elaborate mechanisms to regulate the balance between coagulation and fibrinolysis (7, 29). However, it is noteworthy that, due to endothelial injury in sepsis, large amounts of PAI-1 are released from injuried endothelium, which may tip this balance and cause the fibrinolytic system to shut down (29). Mei et al. (10) conducted a multi-center, prospective and observational study, and demonstrated that TAT, t-PAIC, and soluble thrombomodulin (TM) increased in patients with DIC in adult sepsis, but not PIC, demonstrating sepsis patients' coagulation activation, and fibrinolytic inhibition and endothelial injury. In our cohort, serum levels of PIC increased in pediatrics with severe sepsis, and well correlated with disease severity, in addition, PIC was a strong predictor for severe sepsis in our pediatric patients when explored by univariate logistic regression analysis, but it was not significant in the multivariate model, which could be partially interpreted as the shut-down of fibrinolytic system in sepsis. t-PAIC is a marker of endothelial injury and fibrinolysis activation, which is formed through the combination of tissue plasminogen activator and PAI-1, and is thought to be related with organ failure due to extensive microthrombosis (14). Zhong and her colleagues (15) retrospectively analyzed 311 adults with sepsis who were admitted to the ICU, and found that serum t-PAIC levels are highly correlated with the severity of septic shock and may be an independent risk factor for this condition. Li et al. (30) investigated the clinical utility of TAT, PIC, t-PAIC and soluble TM in pediatric sepsis and pediatrics sepsis-induced coagulopathy (pSIC), and demonstrated that only t-PAIC among these four biomarkers increased in children with pSIC, which suggested t-PAIC, the marker of endothelial injury and fibrinolysis activation, played a crucial role in the pathogenesis of pSIC in pediatric sepsis. Tang et al. (31) enrolled 813 consecutive patients entering the emergency department, with markedly elevated levels of D-dimer (above 5.0 µg/ml, 10 times of the upper normal limit), and identified that sepsis patients had the highest levels of TM and PAI-1, when compared with other types of emergency patients, such as tumour, venous thromboembolism (VTE), trauma, artery dissection and stroke, which suggested that sepsis patients had significant endothelial injury and fibrinolytic inhibition. On the basis of previous researches, our study revealed that t-PAIC also markedly increased in pediatrics with severe sepsis, however, endothelial cell damage, rather than hyperfibrinolysis, was the primary cause of the elevated t-PAIC levels (15, 22). Furthermore, as a matter of fact, extensive microthrombosis led to multiple organ dysfunction in patients with severe sepsis, especially with septic shock primarily owing to fibrinolysis shutdown (32). In addition to this, our results suggested that t-PAIC, along with TAT, may be valuable biomarkers for predicting severe sepsis in pediatrics.

Last but not the least, we have noticed that the definition of pediatric sepsis has recently been updated, and the new guideline emphasized host organ dysfunction, instead of SIRS, caused by infection, including dysfunction of the respiratory, cardiovascular, coagulation, and/or neurological systems (2), which was more closer to the criteria of sepsis 3.0 for adults (33). Collectively, the new diagnostic criteria for pediatric sepsis, which was based on Phoenix Sepsis Score, was more specific for the identification of pediatric sepsis and may have potential value in predicting death. But also, something concerned us was that due to some parameters not availability to easily obtain, especially in remote area like China, a fairly large number of pediatric sepsis patients would be diagnosed delayed, which may exacerbate this fatal syndrome. Meanwhile, the old definition of pediatric sepsis highlighted SIRS caused by infection (1), which was more sensitive for the recognition of pediatric sepsis, in addition, the components of SIRS criteria were more easily to obtain in primary health care institutions or even at home, enabling more probability of early intervention and treatment. Generally speaking on the whole, although the definition of pediatric sepsis had been updated, considering the medical condition of pediatric sepsis in the real world, our research based on the old guideline is still of great practical significance.

Some potential limitations in our study should be mentioned. First, this was a single center, observational study with a relatively small sample size, a sizable validation research is warranted to confirm our findings. Second, we only analyzed the serum levels of TAT, PIC and t-PAIC on the initial day of pediatric sepsis diagnosis, considering that coagulopathy was ongoing and throughout the whole course of the disease, dynamic monitoring of these biomarkers will reveal more information. Third, all tested coagulation markers are unspecific for sepsis, and are also impaired by other acute coagulation challenges, although we had tried our best to exclude these cases in order to minimize the impact of the factors. Fourth, not all predictive parameters are used routinely in most hospitals, although the fully automated analyzers have already been of advent, which would limit the clinical application among pediatrics. Fifth, a multi-parameter prediction model that incorporates coagulation markers together with other factors might potentially offer superior predictive power for severe sepsis in pediatrics.

## Conclusion

Our study provides the novel evidence that TAT, PIC and t-PAIC could serve as biomarkers for predicting severe sepsis, and correlated with disease severity in pediatrics, what's more, serum levels of TAT and t-PAIC may be independent risk factors for pediatric severe sepsis.

## Data Availability

The original contributions presented in the study are included in the article/Supplementary Material, further inquiries can be directed to the corresponding author.
